# Chronic Lithium Toxicity in Old Age Patients Taking Angiotensin Receptor Blocker: A Case Report

**DOI:** 10.31729/jnma.8346

**Published:** 2023-11-30

**Authors:** Asim Pandey, Kunjan Khanal, Anushree Jha, Prava Basnet, Dipesh Bhattarai

**Affiliations:** 1 Norvic International Hospital, Thapathali, Kathmandu, Nepal; 2 Green City Hospital Pvt Ltd, Basundhara, Kathmandu, Nepal; 3 Kathmandu Cancer Center, Bhaktapur, Nepal; 4 Hebei Medical University, Shijiazhuang, Hebei, China; 5 Department of Psychiatry, Green City Hospital, Basundhara, Nepal

**Keywords:** *angiotensin receptor blocker*, *bipolar disorder*, *case reports*, *lithium*, *telmisartan*

## Abstract

Chronic lithium toxicity is a potentially serious side effect on patients taking lithium for a prolonged period with the diagnosis of mood disorders. The toxicity is even higher in patients taking drugs that interfere with the metabolism of lithium like angiotensin receptor blockers and in older patients with reduced kidney function. In this report, we present the case of a 62-year-old woman who presented to the emergency department with symptoms including loose stools, generalised body weakness, slurred speech, coarse hand tremors, and dystonia persisting for fifteen days. She had been under lithium therapy for bipolar type 1 disorder for 15 years before experiencing these symptoms, which emerged shortly after the addition of telmisartan (angiotensinogen receptor blocker) for hypertension.

## INTRODUCTION

Lithium is an inorganic element and is found naturally in the form of salt. After the discovery of lithium in the mid-20th century, its use as a first-line treatment for maintenance therapy for bipolar disorder is of immense value.^1^ Although of its effective use in mood disorders, lithium is associated with various acute and chronic toxicities. Neurologic effects are more common with chronic lithium intoxication which includes coarse tremor, slurred speech, dystonia and ataxia along with altered consciousness whereas gastrointestinal side effects are more common with acute toxicity. Impaired urine concentrating ability, nephrogenic diabetes insipidus, and nephrotic syndrome are well-known renal complications of chronic lithium toxicity.2

## CASE REPORT

A 62-year-old woman and a known case of type 1 bipolar disorder presented to the tertiary care centre with chief complaints of loose stool, generalised weakness, jerky movements of the head, dysarthria, coarse hand tremors and slurred speech for fifteen days duration. She was unable to walk and perform her daily life activities. She has had a past medical history of bipolar type 1 disorder for 15 years and was prescribed lithium 600 mg twice a day. She is a newly diagnosed case of hypertension and has been under telmisartan 40 mg (angiotensin receptor blocker) for fifteen days.

She was referred from a primary health clinic and was examined in the emergency of a tertiary care hospital. On arrival at the emergency (ER), her vitals were normal except for raised blood pressure. Skin examination showed decreased skin turgor owing to chronic watery diarrhoea, six times per day, non-bloody and non-mucoid. On clinical examination, she had coarse tremors of extremities, sustained muscle contractions of the neck and generalised weakness. On neurological examination, she had slurring of speech and was disoriented to time. An urgent head computed tomography (CT) scan of the head showed no structural abnormalities. Magnetic resonance imaging (MRI) done after a week of hospital admission was normal. All the necessary basic investigations like complete blood count, liver and renal function test, and urine electrolytes were ordered along with stool culture, stool ova and parasites as well as thyroid function test. Serum lithium concentration was also ordered. Reports after a day of admission showed no abnormalities except microcytic, hypochromic anaemia and raised serum lithium level. The lithium level at the time of presentation was 1.55 mmol/l. The thyroid function test was within the reference range. The renal function test was within a normal range except for a slight increase in serum urea concentration. Serum vitamin D concentration was below the reference range.

The treatment was then initiated with a multidisciplinary team involving psychiatry, general medicine and cardiology. Lithium 600 mg and telmisartan 40 mg were discontinued and was replaced by olanzapine 0.5 mg and amlodipine 5 mg. IV fluid therapy with normal saline and vitamin D along with calcium therapy was begun and serum lithium concentration was ordered every second day. Serum electrolytes like potassium and sodium, renal function tests and ECG were monitored every 12 hours. The symptoms of slurring of speech and disorientation gradually recovered within a week of therapy initiation. Serum lithium concentration was on a decreasing trend and was 0.46 mmol/l after a week of supportive therapy initiation and lithium discontinuation. Furthermore, the episodes of loose stools gradually subsided and the patient was able to resume daily life activities.

The patient was discharged after nine days of hospital admission and was advised to follow up after a week. Olanzapine 0.5 mg therapy was substituted for lithium for bipolar disorder and amlodipine 5 mg was substituted for telmisartan. After a week following discharge, the patient's bipolar symptoms were adequately controlled and neurological as well as diarrhoea symptoms subsided. She was then advised to follow up after a month.

## DISCUSSION

Lithium therapy is the first choice of drug for the prophylactic treatment and management of recurrent bipolar illness, and manic and depressive episodes.^2,3^ At a neuronal level, lithium decreases the excitatory but increases inhibitory neurotransmitters; however, multiple complex involvements of neurotransmitters are involved to achieve homeostasis.^4^ The incidence of lithium toxicity based on a cohort study was found to be 0.01 per patient-year.^5^

The pathophysiology behind lithium toxicity involves increased intake and/or decreased excretion.^6^ The serum lithium levels can be affected by the use of drugs like angiotensin receptor blockers (ARB) and angiotensinogen converting enzyme inhibitors (ACE) by decreasing the excretion of lithium.^7^ There are various side effects of lithium therapy including neurologic effects like coarse tremor, hyperreflexia, and nystagmus; renal toxicity like nephrogenic diabetes insipidus, sodium losing nephritis, nephrotic syndrome; gastrointestinal side effects like chronic diarrhoea, vomiting and endocrine side effects like hypothyroidism.^6^ The patient was under lithium therapy for fifteen years and developed side effects resembling chronic lithium toxicity. Though the patient had only a mild increment in the toxic dose of lithium level, symptoms may be more severe even with serum lithium levels close to 1.5 mEq/l due to higher lithium concentration in the brain with the use of lithium for a prolonged period.^8^ The use of telmisartan hastened the side effect of lithium therapy which was demonstrated by the recovery of patient symptoms after discontinuation of offending drugs. Aggressive fluid therapy and discontinuation of the offending drugs are the mainstay of treatment. Hemodialysis may be warranted if serum lithium level is greater than 5 mEq/l on presentation or greater than 4 mEq/l with altered kidney function.^9^

Although chronic lithium toxicity can occur in patients who are dependent on the medication, the risk of toxicity can be minimized by avoiding the drugs that interact with lithium metabolism.^7^ Adequate hydration, patient education on the side effects of lithium therapy and health care awareness are of utmost importance in reducing the chronic side effects on the patient with lithium therapy. Unintended lithium toxicity can occur in the elderly despite lower serum lithium concentration and requires lower doses to achieve similar serum concentrations as those in younger adults.^10,11^ Older patients require a dose of lithium 31% lower than those aged <50 years. 11 Therefore, prescription drugs like lithium should be given special consideration in the elderly population and neurotoxicity should be suspected at serum lithium levels which are considered therapeutic in younger adults.^10^
